# Effects of bedside team-based learning on pediatric clinical practice in Chinese medical students

**DOI:** 10.1186/s12909-022-03328-4

**Published:** 2022-04-11

**Authors:** Jie Gong, Junfeng Du, Jinjin Hao, Lei Li

**Affiliations:** 1 The Clinical Skills Center, The First Clinical College, Union Hospital, Tongji Medical College, Huazhong University of Science and Technology, Wuhan, 430022 China; 2 Department of Plastic Surgery, Liyuan Hospital, Tongji Medical College, Huazhong University of Science and Technology, Wuhan, 430077 China; 3 Department of Pediatrics, Union Hospital, Tongji Medical College, Huazhong University of Science and Technology, Wuhan, 430022 China; 4 Department of Pediatrics, Jingshan People’s Hospital, Jingshan, 431800 China

**Keywords:** Pediatrics, Team-based learning, Bedside teaching, Active learning, Counseling skills, Clinical reasoning

## Abstract

**Background:**

Bedside teaching is a primary educational tool to improve the clinical practice of medical students. As a new medical pedagogical approach, team-based learning (TBL) is gradually being integrated into Chinese medical education programmes to promote clinical reasoning, knowledge application, teamwork and collaboration. The aim of this controlled study is to investigate the effects of TBL on pediatric bedside teaching in medical students.

**Methods:**

Thirty medical students in pediatric clinical practice were randomly assigned to an intervention and a control group. Students in the intervention group exposed bedside teaching activity with TBL while students in the control group received traditional bedside teaching. Teaching for the two groups was conducted biweekly, and the same clinical cases were selected for both groups with the same instructors. After six months of clinical practice, the differences of learning outcomes between the two groups were compared through assessments by computer-based case simulations (CCS) and mini-Clinical Evaluation Exercise (mini-CEX). Student feedback following completion of bedside teaching was collected by questionnaire.

**Results:**

The CCS scores in the intervention group were significantly higher than that in the control group (*p* < 0.05). The mini-CEX results showed that clinical judgment and counseling skills of the intervention group were higher than those in the control group (*p* < 0.01). Medical interviewing skills and overall clinical competence in the intervention group were better than those in the control group (*p* < 0.05). In the questionnaire survey, students in the intervention group believed that bedside teaching activity with TBL could promote active learning ability, improve counseling skills and strengthen teamwork.

**Conclusions:**

Application of TBL in bedside teaching not only enhanced clinical practice skills among medical students but also improved their clinical reasoning and counseling skills.

## Background

Currently, it is widely believed that China is facing an evident shortage of pediatricians [1, 2]. It takes years to train a qualified pediatrician featured with accurate perception and judgment, enabling to work effectively with children and their adult guardians. Managing the fear, vulnerability, and anxiety that parents feel when their children aren’t feeling well requires excellent communication and empathy from health care providers. Thus, an effective bedside teaching is particularly important in accelerating integrated understanding of basic and clinical sciences in medical students. Bedside teaching has been seen as one of the ideal clinical teaching modalities, in which history taking and physical examination skills, together with professional attitude, can be combined to provide medical students with a holistic improvement in clinical competency [3]. Traditional bedside teaching in China is a teacher-centered instruction model, failing to address students' learning difficulties, so students are unable to obtain personalized teaching. Considering the importance of bedside teaching to clinical competence of physicians, a need for exploring new practical, bedside teaching methods still exists since medical students have been observed to be lack of motivation to learn and problem-solving ability to participate in the rounds effectively [4]. We believe a student-centered bedside teaching model is desirable and feasible, offering opportunities for students to actively participate in bedside practice teaching and achieve experiential learning.

The concept of team-based learning (TBL) was proposed as a new problem-based, active learning model in 2002 [5] that could retain the educational strengths of problem-based learning (PBL) albeit in a more efficient way [6]. Studies have shown that TBL generates interest among students, encourages them to prepare prior to the class and to work in a team, and boosts their critical thinking abilities, which are associated with their problem-solving ability and academic performance [7].There is growing evidence that TBL in medical education is effective in improving students’ knowledge, attitudes, academic performance, achieving learning goals, as well as improving satisfaction towards the learning process for both students and faculty members [8–10]. Interactive evaluations among students participating in TBL can stimulate individual team members contributing to the team and achieving coordination with each other. TBL can also enhance students’ awareness towards the emotional and attitudinal changes of others, thus playing a positive role in promoting empathy [11].

Although there are extensive empirical studies on active learning strategies [12, 13], the effects of TBL on bedside teaching of pediatrics to improve medical students’ clinical competence are not well studied. Given the knowledge gap and the need to devise effective training modules for pediatricians, this study aimed to compare the effects of TBL versus traditional education on knowledge transfer, clinical performance and problem-solving abilities of medical students in a pediatric bedside teaching setting in China.

## Methods

### Study design

We designed the study to evaluate the effects of TBL teaching versus traditional teaching at the bedside by comparing learning outcomes among medical students in the intervention group and control group. Thirty fourth-year medical students, who agreed to participate voluntarily in our study, were enrolled and randomly allocated to one of the two groups: the intervention group received TBL bedside teaching while the control group received traditional bedside teaching biweekly.

During pediatric bedside practice, students would encounter patients with common, frequently occurring diseases, including bronchopneumonia, Kawasaki disease, allergic purpura, hydrothorax, unknown fever, convulsions, bleeding, neonatal jaundice and shortness of breath. An informed consent was obtained from parents of these pediatric patients prior to each teaching activities. Two full-time senior pediatric faculty members (LL and JJH), who had received standardized training in TBL and bedside teaching, were engaged to teach both groups. In the intervention group, the teachers carried out TBL in bedside teaching of pediatrics. LL was responsible for issuing the learning materials prior to the teaching activities, compiling exercises and collecting the students’ feedback of TBL in bedside teaching. JJH mainly selected clinical cases, supervised the bedside teaching and provided feedback or guidance to students.

#### Teaching arrangement for intervention group

Participants in the intervention group were randomly allocated to three subgroups with five students per subgroup. TBL for intervention group consisted of three phases: advance preparation, readiness assurance and application at the bedside.

### TBL preparation

According to the teaching contents, teachers established the learning objectives and determined the learning scopes. Two days before each intervention, we established a teacher-student communication group by using teaching management application software on the mobile phone and sent basic conditions of related cases, micro-lectures of relevant theoretical knowledge and other materials to the students in the intervention group. The students collected related information and familiarized themselves with the knowledge individually. Adequate pre-class preparation plays an important role in ensuring TBL discussions as well as personal and team growth [14].

### TBL readiness assurance

Prior to class, students would receive the individual Readiness Assessment Test (iRAT) [15] comprising 10 multiple-choice questions, which evaluated their TBL preparation and ensured the implementation of upcoming courses. After all students completed and submitted their iRAT responses, the identical quiz questions were handed out to the students for completion as a team, consulting with each other to determine a consensus answer. Upon obtaining correct answers, the team Readiness Assurance Test (tRAT) [15] was completed. Following the tRAT, the teachers analyzed the answers given by each student and allowed them to present their argument regarding their case. The assessment phase is a major component of TBL, which motivates students to prepare prior to the class and promotes team cohesion.

### TBL application at the bedside

Within each subgroup of the intervention group, bedside practice tasks were divided into five steps. Each step was taken one-by-one and was led by one subgroup member. The assignment of each student's task is shown in Table 1. In a consulting room, Student 1 took medical history from the children’s parents; Student 2 carried out the physical examination at the bedside; Student 3 summarized the medical history and completed clinical reasoning, and Student 4 made a working diagnosis and therapeutic strategy in a conference room. Finally, Student 5 communicated with the parents for delivering medical information in a consulting room.

**Table 1:** Tasks division in TBL bedside teaching activities

Step	Task	Location	Participant
1	History taking	Consulting room	Student 1
2	Physical examination	Bedside	Student 2
3	Clinical reasoning	Conference room	Student 3
4	Decision making	Conference room	Student 4
5	Communication	Consulting room	Student 5

Students 1 and 5 completed the task supervised by teachers. The other four students observed the conversation between the student and parents on live video in an adjunct room. Without disturbing the students, the teachers offered appropriate guidance and supplements after the process of conversation. During Step 2–4, the teachers gave a guidance to students for making suggestions and supplements at the end of each step.

Following TBL bedside teaching activities, all the students came together and debriefed with each other and teachers. Firstly, all the students performed self-evaluation of TBL bedside teaching procedure individually, and then the teachers guided all the students with feedback and reflection. Finally, the teachers summarized key points and commented on students’ performance.

#### Teaching arrangement for control group

**Fig. 1:**
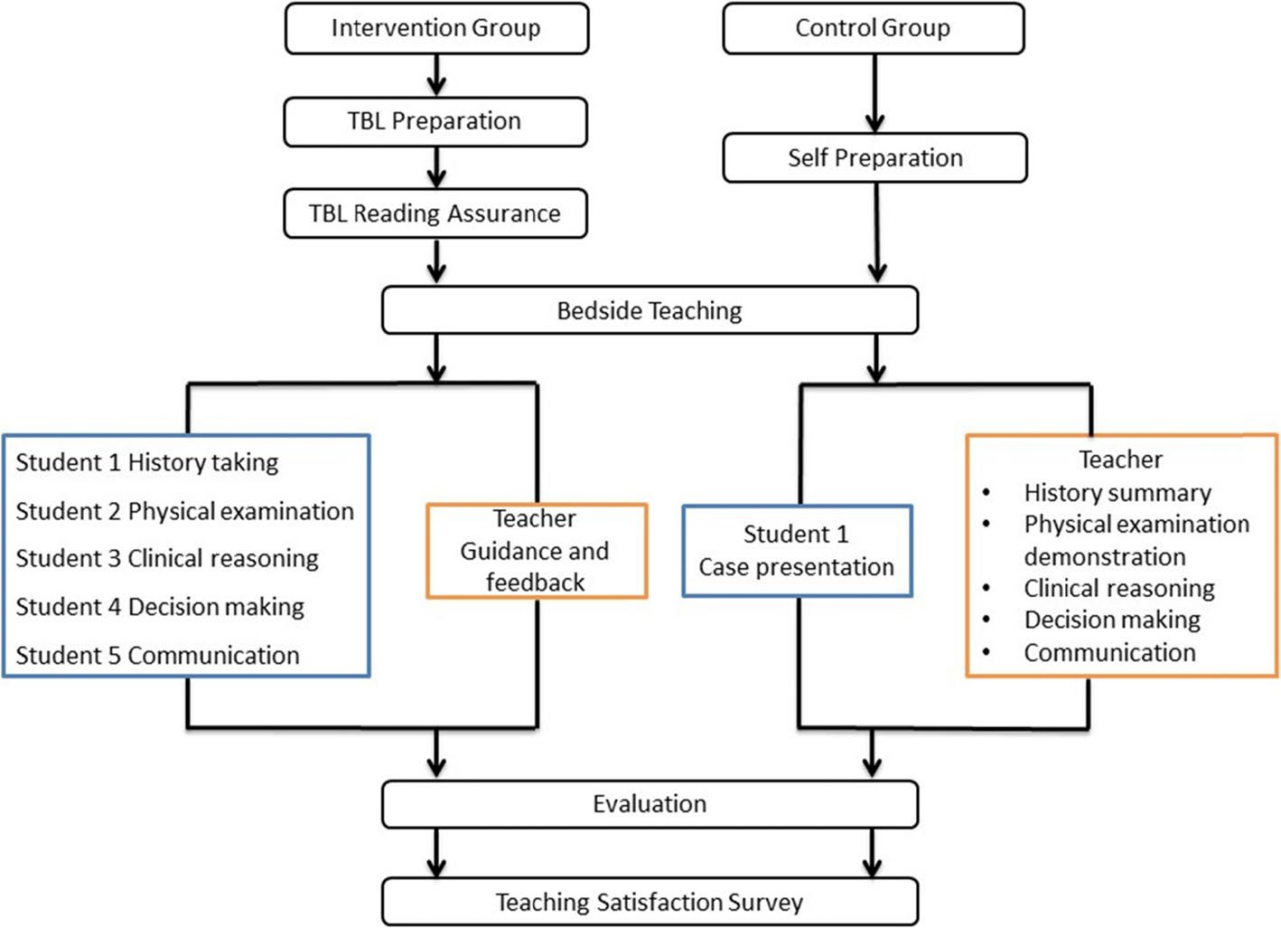
Flow chart of bedside teaching with team-based learning and traditional teaching rounds

Students in the control group were randomly allocated to three subgroups with five students per subgroup. Instead of TBL, the students participated in traditional teaching rounds. The students were informed with selected cases and previewed the related information independently before the teaching rounds. During the teaching rounds, one student presented case findings to the group while the teachers demonstrated standardized physical examination, probed for underlying reasoning, proposed the diagnosis and therapeutic strategies, communicated with parents and delivered related medical information to them. Teachers kept interacting with students during the whole process, and confirmed or corrected the case findings reported by the student. Figure 1 shows the flow chart for the two groups in bedside teaching.

### Outcome measures

#### Examination of cognitive knowledge

Cognitive knowledge involved in history taking, physical examination, clinical reasoning and decision making was assessed via computer-based case simulations (CCS) [16]. Through interactive responses between students and computers, students simulated doctors to treat patients in virtual medical situations. The CCS examination was set at 60 min with a maximum score of 100 points with unified marking by the computer after submission. The scoring domains include professional attitude, clinical practical skills, learning ability, counseling skills, clinical reasoning and time management. In addition, a roadmap of students’ diagnostic processes were created to visualize the clinical reasoning aspect to help students’ self-feedback.

### Examination of clinical performance

The examination of clinical performances was based on mini-Clinical Evaluation Exercise (mini-CEX) in the form of clinical cases at bedside [17]. The marks of the mini-CEX tool were given for seven items covering medical interviewing skills, physical examination skills, humanistic qualities, clinical judgment, counseling skills, organization/efficiency, and overall clinical competence with 9 points for each item. The higher the score, the better the performance. Actual assessments using mini-CEX were conducted by JG, who was not involved in the bedside teaching to be blinded from the study group allocation of the students. JG was a qualified evaluator who received a training of mini-CEX, and passed the accreditation examination assigned by our college.

### Students’ satisfaction survey

An anonymous survey was conducted with an online questionnaire [18] to investigate the satisfaction of students who participated in this teaching activities. Questionnaires were designed using WJX online survey platform (https://www.wjx.cn) [19], in which the items covered six main areas: students’ overall satisfaction, active learning attitudes, students’ clinical skills, the degree of understanding in theoretical knowledge, counseling skills and teamwork. The survey used a five-point Likert, with which students or teachers were asked to rank their responses: 1 (strongly disagree); 2 (disagree); 3 (neutral); 4 (agree) and 5 (strongly agree). Their responses were captured anonymously in WJX, and the data obtained from the questionnaires was analyzed by Cronbach’s alpha test to determine the internal consistency of responses.

### Ethical approval

This study was approved by the Research Ethics Committee at Tongji Medical College of Huazhong University of Science and Technology, and conducted according to the Helsinki Declaration.

### Data analysis

All data were analyzed using the SPSS (Version 18.0). Data were expressed as mean ± standard deviation. *t* test or chi-square test between two groups was adopted, and *P* less than 0.05 was considered as statistically significant.

## Results

### Participants

The study was conducted over a six-month period between March 2019 and August 2019. None of enrolled medical students dropped out during the implementation of this study. Demographic information of all the participants were listed in Table 2. No statistically significant differences was observed between control group and intervention group in terms of age, gender, and theoretical examination scores in the fourth year of medical college.

**Table 2:** Demographic characteristics of students in two groups

Group	Age	Gender	Theoretical Examination Scores
Intervention Group (*n* = 15)	22.53 ± 0.23	Male: *n* = 8 Female: *n* = 7	84.13 ± 2.25
Control Group (*n* = 15)	22.29 ± 0.24	Male: *n* = 9 Female: *n* = 6	83.67 ± 3.17
	*t* = 1.012	*χ*^*2*^ = 0.136	*t* = 0.207
*p*	0.320	0.713	0.838

### Comparison of CCS and mini-CEX results in two groups

Results of CCS and mini-CEX examination between control group and intervention group were compared to evaluate the students’ clinical skills in pediatrics respectively. The results of CCS in the intervention group were significantly higher than that in the control group (*t* = 3.216, *p* = 0.0033) (Table 3).

**Table 3:** Comparison of CCS and mini-CEX results between the two groups (Mean ± SD, points)

Group	CCS Scores	mini-CEX						
		Medical interviewing skills	Physical examination skills	Humanistic qualities	Clinical judgment	Counseling skills	Organization/efficiency	Overall clinical competence
Intervention group (*n *= 15)	85.73 ± 4.200	7.067 ± 0.7988	6.200 ± 0.6761	6.067 ± 0.8837	6.933 ± 0.9612	6.800 ± 0.8619	5.933 ± 0.7988	6.067 ± 0.7037
Control group (*n* = 15)	80.13 ± 5.276	6.333 ± 0.8997	5.933 ± 0.8837	5.600 ± 0.9103	5.800 ± 0.8619	5.733 ± 0.8837	5.333 ± 0.8997	5.267 ± 0.7037
*t*	3.216	2.361	0.928	1.425	3.400	3.347	1.931	3.113
*P*	0.0033	0.0254	0.3612	0.1653	0.0020	0.0023	0.0636	0.0042

**Fig. 2:**
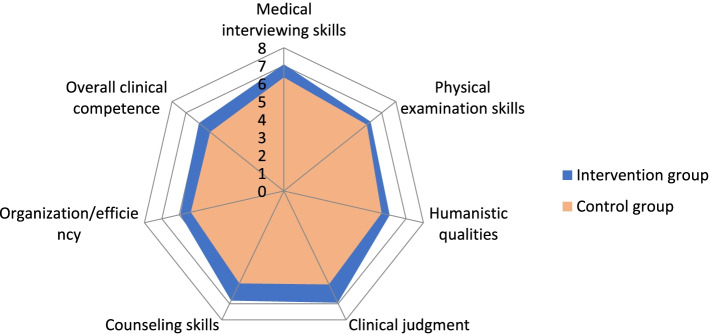
Comparison of clinical comprehensive practical skills between the two groups

We conducted mini-CEX as a clinical assessment method to measure students’ clinical skills, including medical interviewing skills, physical examination skills, humanistic qualities, clinical judgment, counseling skills, organization/efficiency, and overall clinical competence. As shown in Table 3 and Fig. 2, clinical judgment and counseling skills of the intervention group were higher than those in the control group (*t* = 3.400, *p* = 0.0020; *t* = 3.347, *p* = 0.0023), and medical interviewing skills and overall clinical competence in the intervention group were better than those in the control group (*t* = 2.361, *p* = 0.0254*; t* = 3.113*, p* = 0.0042). However, there were no significant differences in physical examination, humanistic qualities and organization between the two groups (*t* = 0.928, *p* = 0.3612; *t* = 1.425, *p* = 0.1653; *t* = 1.931, *p* = 0.0636).

### Comparison of students’ satisfaction in two groups

For 30 questionnaires received (response rate 100%), Cronbach’s α coefficient was 0.81, which implied the survey had good internal consistency and reliability. Compared to the control group, students’ satisfaction in the intervention group was significantly higher than that of the control group: overall satisfaction (*t* = 2.497, *p* = 0.0187), stimulation of active learning attitude (*t* = 3.568, *p* = 0.0013), improvement of clinical skills (*t* = 2.928, *p* = 0.0067), facilitation of counseling skills (*t* = 4.291, *p* = 0.0002) and enhancement of teamwork (*t* = 3.674, *p* = 0.0010) were higher than those of the control group. There was no significant difference in mastering theoretical knowledge between the two groups (Table 4).

**Table 4:** Comparison of teaching satisfaction between two groups (Mean ± SD)

Items surveyed	Intervention group (*n* = 15)	Control group (*n* = 15)	*t*	*p*
Overall satisfaction	4.600 ± 0.5071	4.133 ± 0.5164	2.497	0.0187
Stimulate active learning	4.667 ± 0.4880	4.000 ± 0.5345	3.568	0.0013
Master theoretical knowledge	4.467 ± 0.5164	4.200 ± 0.4140	1.560	0.1299
Improve clinical skills	4.600 ± 0.5071	4.133 ± 0.3519	2.928	0.0067
Facilitate counseling skills	4.533 ± 0.5164	3.800 ± 0.4140	4.291	0.0002
Emphasize more on teamwork	4.600 ± 0.5071	4.000 ± 0.3780	3.674	0.0010

## Discussion

Rapid development of educational environments has influenced medical education considerably [10] and traditional training models can no longer adequately meet the students’ learning needs. Currently, competence-oriented teaching is recognized to be good paradigms in medical education. Based on Miller’s pyramid theory, practice by doing is an effective learning pathway for a medical practitioner to improve clinical skills [20]. Essentially, competence-oriented teaching is one of student-centered teaching approaches, which could develop the knowledge, skills and attitudes of medical students.

However, traditional bedside teaching in China emphasizes actions or behaviors demonstrated by teachers rather than what students actually learnt, thus benefiting few students. Despite more than one year of internship, the students were still proved to be deficient in clinical skills, leading to their inability to perform basic medical procedures independently. Based on observation in our clinical teaching activities, we discovered that students have various difficulties in clinical practice learning. These students in difficulties presented poor communication with patients, lack of skills in recognizing signs and making a diagnosis, in accordance with previous study [21]. These above problems indicate that clinical competence of students has not been improved in the traditional bedside teaching, and lack of active and personal practice might be one of the important reasons. Thus, a new bedside teaching approach is explored to facilitate students to be active participative learners.

Active learning methods have become increasingly popular in modern medical curricula as a reformation to cultivate the medical talents of active learning ability, innovation and cooperation [22]. Bedside teaching activities are continuously challenged by the call for more evidence-based effective learning methods that actively help medical students to develop critical thinking and problem-solving abilities in clinical practice. As an active learning strategy, TBL has the potential to improve the effectiveness of bedside teaching.

This study demonstrated students in the intervention group scored significantly higher in CCS as compared to the control group, while the results of mini-CEX also showed that medical interviewing skills, clinical judgment, counseling skills and overall clinical competence of the intervention group were significantly better than performance of students in the control group. Our findings suggest that TBL could improve the students’ clinical reasoning and counseling skills. Unlike the traditionally teaching rounds, teachers have shifted roles from a lecturer to a facilitator of learning activities, and students took over the lead roles in the bedside teaching activities. By discussing with students, teachers can provoke a deeper learning among students with clinical problem solving, improve students’ clinical reasoning ability while establishing and maintaining a good learning environment. These findings are in alignment with the result of Currey et al*.* showing that the quality of learning, clinical reasoning, professional development, and satisfaction with team experience were increased via TBL [23]. Compared with traditional teaching modes, the ratio of students to teachers in TBL is high, which has the potential to effectively alleviate the problem of a relative lack of teachers while large number of medical students in one medical education institute are often the case in China [12]. Additionally, TBL could help increase the interaction between teachers and students, improve students’ learning efficacy and give full support to the students’ subjective initiative.

Using questionnaires to investigate the learning outcomes between the two groups, the survey result yields the suggestion that TBL significantly contributed towards improving active learning, counseling skills, clinical reasoning and teamwork among students. We could conclude that TBL can not only stimulate interest in learning, but also enable students to establish a habit of active learning. Additionally, TBL pays more attention to training students to solve problems and cultivates clinical reasoning rather than simply remembering the knowledge itself, which promotes the application of knowledge, a higher level of learning [24]. Studies have shown that students’ oral expression and active communication were improved through history taking, team discussion and conclusion [25]. Similarly, our results strongly suggest that TBL is helpful in improving students' clinical competency, facilitating their clinical reasoning skills and laying a foundation for the cultivation of good counseling skills.

### Limitations

Although this study illustrated that bedside teaching activities with TBL improved active learning, clinical reasoning, counseling skills and overall clinical competence in medical students in pediatrics, we also noted some problems in bedside teaching activities with TBL. Firstly, some shortcomings of TBL include scattered knowledge points and lack of systematical learning. Therefore, teachers should take into account the systemic knowledge of selecting cases, and strengthen the designing of iRAT and tRAT test questions [26]. Secondly, time spent on bedside teaching was identical in two groups. Nevertheless, students in the intervention group reviewed the theoretical knowledge related to cases under the guidance of teachers, and they conducted interactive learning with teachers prior to bedside teaching activities. Although students in the control group were also required to review relevant knowledge in advance, the duration of autonomous learning was no guarantee and their learning outcomes were not tested before the teaching activities, contributing to differences of extracurricular time spent on independent learning between the two groups. Thus, participants with new bedside teaching approach may have spent more time on their learning, which is an influencing factor in this study. To reduce possible confounding factors, future studies should consider controlling total time spent on tasks and processing of teaching formats for both TBL participants and non-participants. Lastly, clinical engagement presents challenges for bedside teaching, teaching activities is usually arranged to avoid the morning working hours when medical procedures are concentrated.

It’s worth noting that TBL posed a new challenge to teaching skills from the perspective of teachers, thus they were required to improve teaching design and optimize teaching resources. Then, teachers need to make full preparations before teaching activities, including providing learning resources, selecting appropriate cases, and making teaching schedules, so as to promise a smooth implementation of TBL bedside teaching. Meanwhile, teachers who participate in TBL bedside teaching are relatively fixed and receive regular training on teaching methods to ensure the uniformity and sustainability of the teaching process.

## Conclusion

In this study we proposed and demonstrated the effectiveness of a TBL-enabled bedside teaching approach that provoke active learning, and improve students’ clinical reasoning, counseling skills and overall clinical competence among medical students in a pediatric setting. Clinical performance was tested comprehensively using CCS and mini-CEX, and the integration of virtual simulation in CCS with the practice on patients enabled students to master and understand the whole processes of diagnosis and treatment. Active team discussion and feedback strategies used in TBL were effective in obtaining positive learning outcomes. More research is recommended with medical students of other disciplines to confirm findings of this study and to evaluate the potential positive effects of TBL. Reasonable application of TBL teaching mode is believed to bring a training system of pediatric clinical practice to a new level.

## Data Availability

The datasets used and analyzed during the current study can be provided by the first author on a reasonable request.

## Abbreviations

TBL: Team-Based Learning
CCS: Computer-based Case Simulations
mini-CEX: Mini-Clinical Evaluation Exercise
iRAT: Individual Readiness Assessment Test
tRAT: Team Readiness Assessment Test
